# A systematic review of special events to promote breast, cervical and colorectal cancer screening in the United States

**DOI:** 10.1186/1471-2458-14-274

**Published:** 2014-03-24

**Authors:** Cam Escoffery, Kirsten C Rodgers, Michelle C Kegler, Regine Haardörfer, David H Howard, Shuting Liang, Erika Pinsker, Katherine B Roland, Jennifer D Allen, Marcia G Ory, Roshan Bastani, Maria E Fernandez, Betsy C Risendal, Theresa L Byrd, Gloria D Coronado

**Affiliations:** 1 Department of Behavioral Sciences and Health Education, Rollins School of Public Health, 1518 Clifton Road, Atlanta, GA, USA; 2 Department of Health Policy and Management, Rollins School of Public Health, 1518 Clifton Road, Atlanta, GA, USA; 3 Division of Epidemiology and Community Health, University of Minnesota, Minneapolis, MN, USA; 4 Centers for Disease Control and Prevention, Division of Cancer Prevention and Control, Epidemiology and Applied Research Branch, 4770 Buford Hwy NE, MS K-55 Atlanta, GA, USA; 5 Tufts University, 112 Packard Avenue, Medford, MA, USA; 6 Department of Social and Behavioral Health, School of Rural Public Health, Texas A&M University, 1266 TAMU, College Station, TX, USA; 7 Department of Health Policy and Management, University of California at Los Angeles, 650 Charles E. Young Sr. Dr. S. A2-125 CHS, Box 956900, Los Angeles, CA, USA; 8 Department of Behavioral Sciences, University of Texas-Houston, 7000 Fannin St., Suite 2558, Houston, TX, USA; 9 Department of Community and Behavioral Health, University of Colorado, 13001 East 17th Bldg 500, Office Number W6116, Aurora, CO, USA; 10 Texas Tech Paul L Foster School of Medicine, 1100 North Stanton, Suite 110, El Paso, TX, USA; 11 Kaiser Permanente Center for Cancer Research, 3800 N. Interstate Ave., Portland, OR, USA

**Keywords:** Cancer screening, Early detection of cancer, Health promotion, Community health education, Breast neoplasms, Cervical neoplasms, Colorectal neoplasms

## Abstract

**Background:**

Special events are common community-based strategies for health promotion. This paper presents findings from a systematic literature review on the impact of special events to promote breast, cervical or colorectal cancer education and screening.

**Methods:**

Articles in English that focused on special events involving breast, cervical, and/or colorectal cancer conducted in the U.S. and published between January 1990 and December 2011 were identified from seven databases: Ovid, Web of Science, CINAHL, PsycINFO, Sociological Abstract, Cochrane Libraries, and EconLit. Study inclusion and data extraction were independently validated by two researchers.

**Results:**

Of the 20 articles selected for screening out of 1,409, ten articles on special events reported outcome data. Five types of special events were found: health fairs, parties, cultural events, special days, and plays. Many focused on breast cancer only, or in combination with other cancers. Reach ranged from 50–1732 participants. All special events used at least one evidence-based strategy suggested by the Community Guide to Preventive Services, such as small media, one-on-one education, and reducing structural barriers. For cancer screening as an outcome of the events, mammography screening rates ranged from 4.8% to 88%, Pap testing was 3.9%, and clinical breast exams ranged from 9.1% to 100%. For colorectal screening, FOBT ranged from 29.4% to 76%, and sigmoidoscopy was 100% at one event. Outcome measures included intentions to get screened, scheduled appointments, uptake of clinical exams, and participation in cancer screening.

**Conclusions:**

Special events found in the review varied and used evidence-based strategies. Screening data suggest that some special events can lead to increases in cancer screening, especially if they provide onsite screening services. However, there is insufficient evidence to demonstrate that special events are effective in increasing cancer screening. The heterogeneity of populations served, event activities, outcome variables assessed, and the reliance on self-report to measure screening limit conclusions. This study highlights the need for further research to determine the effectiveness of special events to increase cancer screening.

## Background

Cancer remains the second leading cause of death in the U.S. in 2011 [1]. This equates to more than half million lives lost each year (both genders and all races combined) [1]. Cancer is not only responsible for a vast proportion of mortality in the United States, but also represents a significant disease burden [1]. Breast cancer is the most prevalent cancer in women, and colorectal cancer is the third most common cancer in both females and males [2]. In 2009, breast, cervical and colorectal cancers combined account for nearly one-quarter of the new cancer cases (female breast- 211,731; uterine cervix- 12,357; colorectal cancer- 66,494 in females and 70,223 in males ) [3] and nearly 20% of expected cancer deaths (female breast- 40,676; uterine cervix- 3,909; colorectal cancer- 25,042 in females and 26,808 in males ) [4].

The U.S. Preventive Task Force recommends population-based screening for colorectal, female breast and cervical cancer, leading to earlier detection and treatment, thereby reducing morbidity and mortality [5-7]. Cancer mortality trends suggest that just over a million cancer deaths were averted between 1991 and 2008, likely attributed to improvements in prevention, early detection through screening, and treatment for common cancers [4]. Further, screening has been linked to the steady decline in the incidence rate of colorectal cancer which began in this same time period [8].

Yet, the full potential of early detection through screening has not been realized. Data from the 2010 National Health Interview Survey shows progress toward achieving *Healthy People 2020* objectives for colorectal cancer screening, but breast and cervical cancer screening did not increase between 2000 and 2010. Overall, screening for breast, cervical, and colorectal cancer remains below *Healthy People 2020* objectives; approximately 41% of adults were not adherent to screening guidelines for colorectal cancer; 27% of women aged 50–74 years had not received a mammogram during the preceding 2 years, and 17% of women aged 21–65 years had not received a Papanicolaou (Pap) test during the preceding 3 years. Screening compliance varied markedly by race, ethnicity, education, availability of healthcare and length of U.S. residence [9].

Various interventions to improve the uptake of recommended cancer screenings have been systematically reviewed and summarized in Cochrane Reviews and CDC’s Guide to Community Preventive Services (The Community Guide). A recent update of The Community Guide found additional evidence for existing recommendations, as well as new intervention strategies identified in nine published systematic reviews [10]. A separate systematic review of intervention trials published between 2004 and 2010 reported similar conclusions [11]. The Community Guide recommends: 1) provider assessment and feedback; 2) client reminders; 3) one-on-one education and 4) small media to increase the uptake of breast, cervical, and colorectal cancer screening and reducing structural barriers for promoting breast and colorectal cancer. Additionally, The Community Guide indicates that sufficient evidence exists to recommend group education and reducing client out-of-pocket costs as effective means for increasing breast cancer screening; currently, evidence is insufficient for the effectiveness of these strategies to improve cervical and colorectal cancer screening. Twenty-eight protocols for evidence-based interventions to improve screening uptake are available on Cancer Control P.L.A.N.E.T website [12], 22 are targeted toward minority and underserved groups.

While none of the systematic reviews, nor any intervention protocols have evaluated the use of “special events” as an effective strategy for improving cancer screening rates for any of the three screening amenable cancers, special events are widely used to promote cancer screening and other health behaviors [13]. Special events are defined as community cultural events, charity walks/runs, receptions/parties, pow-wows, and health fairs, and are routinely conducted by state health departments, community-based organizations and other agencies to disseminate health promotion activities to the community. Special events can serve several basic functions: 1) raising awareness and promoting knowledge regarding health topics, 2) providing referrals to local services, 3) facilitating relationships among organizations; and 4) offering community contact for training of nursing, medical, health education or public health students [14-17]. Benefits of these special events can include attracting large numbers of people who could potentially benefit from health promotion, creating of a sense of partnership around health issues affecting the community, and the provision of education and screening for free or reduced cost [13,18].

Special events for cancer screening are ubiquitous. One-third of Recruitment Coordinators in the National Breast and Cervical Cancer Early Detection Program (NBCCEDP), which funds breast and cervical cancer screening services for 67 states, tribes, tribal organizations, territories and jurisdictions, cite using special event activities as part of their outreach and recruitment efforts for breast and cervical screening [19]. However, there is a dearth of literature regarding the specific objectives of special events, the effectiveness of these events in terms of recruiting people in need of health services or referral to services, and the short- or long-term impact on health behaviors such as screening and repeat screening. Evaluation measures are also lacking.

The purpose of this systematic review was to understand the use and value of special events in promoting cancer screening uptake. It answers the following research questions. What are the interventions or strategies employed in special events? What are outcomes of special events? The review sought to elucidate the types of special events used by state, local or community organizations to promote breast, cervical and colorectal cancer screening, and key intervention strategies employed during the event, as well as to document outcomes of such events. Importantly, as one of the first such reviews, this paper lays the groundwork for an evidence base for the use of special events in cancer screening promotion and uptake.

## Methods

### Project team

An advisory committee consisting of cancer researchers, funders, and practitioners from the CDC’s Division of Cancer Prevention and Control, and the Cancer Prevention and Control Research Network (CPCRN) was formed to guide the review process. Comprised of eight members with expertise in cancer epidemiology, behavioral research, health promotion, and nursing, the committee advised on systematic review methods (inclusion criteria for studies, and database elements to include), and the conceptual framework for the systematic literature review. To assure adherence to standardized procedures for systematic reviews, the project team employed existing guides on systematic reviews for the development and implementation of the study methodology, including the Cochrane Handbook of Systematic Reviews of Health Promotion and Public Health Interventions [20], the Community Guide on Systematic Review [21] and TREND statement [22].

### Conceptual model

**Figure 1:**
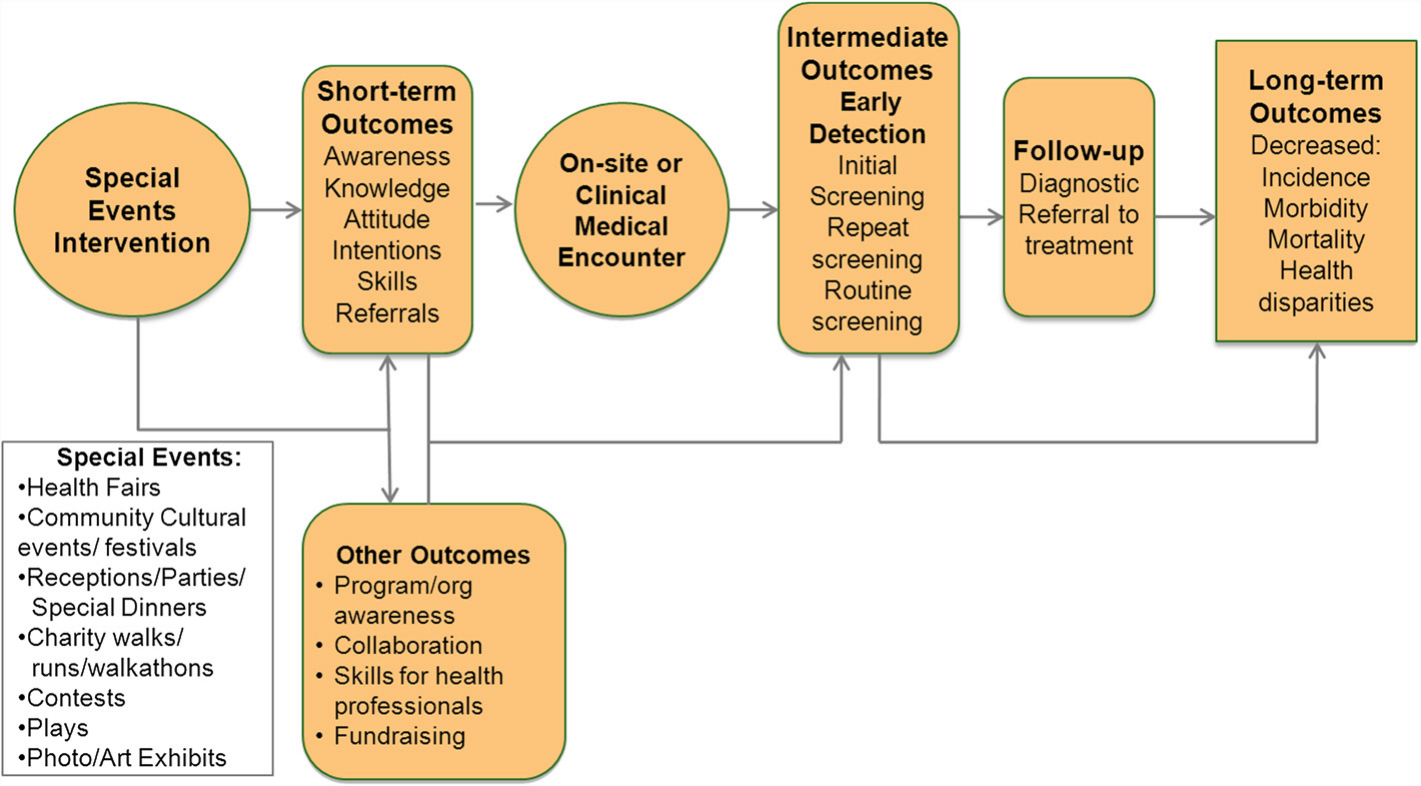
Conceptual model for the use of special events to increase cancer screening.

A conceptual model was developed to show the hypothesized relationships between the special event, determinants of cancer screening behavior, actual screening behavior, and decreased morbidity, mortality and health disparities (Figure 1). Screening determinants and completed screening are two primary outcomes of interest in this review. Short-term outcomes of special events including increased awareness, knowledge, intentions to get screened, and referrals for screening are hypothesized to lead to onsite or clinical medical encounters. The outcome of these clinical visits are expected to result in initial screening, repeat screening, and routine screenings, which lead to an increase in early detection and referral to treatment of disease. Increased screening rates therefore can impact our long term outcomes of decreased incidence, morbidity and mortality caused by breast, cervical, and colorectal cancers. In addition, increasing cancer screening rates through the use of special events has the potential to decrease the health disparities associated with breast, cervical, and colorectal cancers because events’ targeted audiences may be those more affected by the disease and more likely to be uninsured or underinsured.

### Inclusion criteria

The project team created an Eligibility Assessment Checklist to determine which articles would be included for full-article review. The articles were restricted to those: 1) written in English, 2) published from January 1990 to December 2011, 3) examining at least one of the predefined categories of special events, 4) involving cancer screening for breast, cervical, and/or colorectal cancer, 5) including outcome data, and 6) conducted in the United States. Articles were excluded if they did not report on screening determinants (e.g., knowledge about cancer screening, intentions to get screened, making an appointments) or health outcomes (i.e., cancer screening); if the type of cancer or outcomes were not related to breast, cervical or colorectal cancer screening or were not apparent through reviewing the full article; or if the full article was unable to be located after an exhaustive search.

### Search strategy

In February 2012, the study team searched the following databases: Ovid, Web of Science, CINAHL, PsycINFO, Sociological Abstract, EconLit, and Cochrane Libraries. Keywords for special events included: *health fair, cultural event, charity walk/run/walkathon, reception/dinner/gala, play, contest, and art/photo exhibit.* These terms were combined with terms representing different cancer screenings and evaluation. Some general examples include *cancer prevention and control, breast cancer/breast cancer screening, cervical cancer/cervical cancer screening, and colorectal cancer/colorectal cancer screening, evaluation, and cancer screening.* The review team also searched for articles related to cost analyses using Ovid and EconLit. Keywords for the cost analysis were: *cost(s), cost-analysis, cost-effectiveness, cost-utility, or cost-benefit*. These were combined with the keywords developed for special events. The search terms were developed based on MeSH headings and through consultation with a certified health sciences librarian. Manual cross-referencing of reference lists was also completed to ensure the literature search was exhaustive. Relevant articles were downloaded into a reference manager software program that removed duplicate articles identified in the multiple databases. The resulting composite library was then exported into an Excel file for documentation of the abstract review process.

### Screening

**Figure 2:**
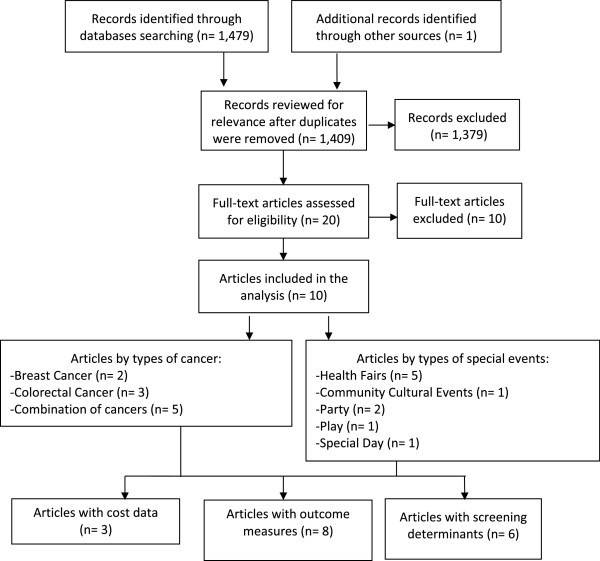
Flow diagram of the systematic review.

The study selection process is illustrated in Figure 2. Two reviewers independently screened the title and abstracts of all citations using an Eligibility Assessment Checklist. Abstracts were classified as relevant, potentially relevant, or not relevant. Relevant abstracts were selected for a full review of the article. Abstracts of potentially relevant articles were further examined independently by the two reviewers. If there was discordance between the two reviewers on the relevancy of an abstract, a third reviewer read the abstract to determine relevancy.

### Data abstraction and analysis

A Data Abstraction Form was used to record information on the article, including title, author(s), publication type, methods, results, reported cost of the event, resources, and barriers to implementation. For each study, we examine bias in the study through documentation of participants (e.g., selection, generalizability), study design, inadequate results reporting and two trained reviewers abstracted the full-text of all relevant articles independently. After both reviewers completed abstraction, consensus on all elements of the abstraction form was reached. A larger team discussed and made final decisions on the few discrepant items. All data were entered into an Access database and descriptive analysis was conducted. To describe common strategies employed, the review team categorized the activities that occurred during the special event into the recommended Community Guide categories for interventions to increase cancer screening [21,23-25]: 1) small media, 2) one-on-one education, 3) group education, 4) reducing structural barriers, and 5) reduced client out-of-pocket costs. The team calculated screening rates by dividing the reported number screened by the number of total participants per special event. Additional file 1: Data Abstraction Form.

## Results

The literature search identified 1,479 articles and additional records identified through other sources yielded 1 article. Of these, the project team screened 1,409 abstracts for eligibility, (after duplicates were removed) of which 1,379 articles did not meet eligibility criteria and were excluded (Figure 2). Many articles were excluded because they either focused on a particular cancer in general and/or were not focused on a special event, or did not have any outcomes data. Twenty articles were selected for full-text review, and of those, 10 articles were determined to be relevant and data were abstracted. Of the ten identified relevant articles on special events, four were published in the 1990s and six studies were in 2000 or later. The ten articles are described in Table 1. Single component articles are those community projects with just the special event, while those community projects that included the special event and other outreach activities were considered multi-component.

**Table 1:** Summary characteristics of included special events

**Single component articles**								
**Author (year)**	**Cancer focus**	**Data collection**	**Participants**	**Sample (N)**	**Special event description**	**Follow-up period**	**Results**	**Cost**
Dolin & Butterfield [26]	Breast and cervical	Post-test only	White women from a mid-eastern U.S. city	67	Type: Health fair	At event	Screening:	Not reported
					Event components:		Scheduled breast and cervical screening appointment:	
					• Group Education and one-on-one education (treatment and control)		Treatment: 41%	
							Control: 25%	
					• Small media (breast self-examination educational information) (treatment)		p-value <.015	
					Referral to screenings			
					Community Guide strategies:^1^ GE, 1on1, SM, RSB			
Englisbe et al. [34]	Breast	Post-event only	Low income, minority women in targeted zip codes in Albany, NY, 63% no prior mammogram	50	Type: Special day	At event	Screening:	$2,500
					Event components:		Mammography: 44 (88%)	
					• Transportation to mammography appointments on Mother’s Day and Valentine’s Day			
					• Educational materials (e.g. totes and t-shirts with health messages)			
					Provide reduced cost mammograms			
					Community Guide strategies: RSB, ROPC, SM			
Fournier et al. [27]	Breast & cervical	Post-event only	Residents of Big Pine Key and Key West, Florida	1279 over 2 yrs	Type: Health fair (offered twice)	At event	Screening:	$7,000
					Event Components:		Mammography (1997):	
					• Free clinical exams: breast and cervical (1996 & 1997)		Total: 34 (4.8%)	
					• Free cancer screening: mammograms (1997)		Clinical Exams (1996–1997):	
							Clinical Breast Exams:	
							133 (10.4%)	
					Patient education		Clinical Female Pelvic Exam (1996–1997): 151 (8.5%)	
					Community Guide strategies: 1 on 1, ROPC RSB			
Resnick [28]	Breast, cervical & colorectal	Post-event only	Residents over 90 years of age (mostly female) living in a continuing-care retirement community (location not specified)	51	Type: Health fair	At event	Screening:	Not reported
					Event components:		Pap Tests: 2 (3.9%)	
					• Clinical exams		Returned FOBT cards: 15 (29.4%)	
					• Cancer screening; breast exams, pap tests, and stools for occult blood test		Clinical Exams:	
					One-on-one education sessions to discuss screening options and chose the relevant screenings		Clinical Breast Exams: 8 (15.7%)	
					Community Guide strategies: ROPC, 1 on 1, RSB			
Gellert et al. [33]	Breast & colorectal	Pre-event and post-event	Native Hawaiians in Molokai, Hawaii	73	Type: Cultural festival	Weekly up to 6 mo	Screening :	Not reported
					Event Components:		Colorectal Cancer (FOBT) (28 men ≥ 50 years old):	
					• Cancer 101 group session		Pre-event: 11 (39%)	
					• One-on-one education		Follow-up: 21 (75%)	
					• Screening: breast exams and colorectal cancers kits		p-value= .002	
					• Follow-up letter at 1 month and up to 6 months phone call to assist with obtaining insurance, scheduling appts., and transportation		Colorectal Cancer (FOBT) (25 women ≥ 50 years old):	
							Pre-event: 9 (36%)	
					Community Guide strategies: ROPC, 1 on1, GE, RSB		Follow-up: 19 (76%)	
							p-value=.002	
							Mammography (38 women ≥ 40 years old):	
							Pre-event: 25 (66%)	
							Follow-up: 32 (84%)	
							p-value=.02	
							Clinical Exams:	
							Clinical Breast Exam:	
							Pre-test: 25 (66%)	
							Post-test: 38 (100%)	
							p-value<.001	
Byrne and Robles-Rodriguez [31]	Breast	Post-event only	Underserved, minority women aged 18–70 in Camden County, New Jersey	568	Type: Party (offered 25 times)	2 days	Screening and Clinical Exams:	Not reported
					Event Components:	After the event	Mammograms and clinical breast exams:^2^ 301	
					• Group education			
					Referrals for screening			
					Community Guide strategies: GE, RSB			
Cueva et al. [35]	Colorectal	Post-event only	Alaska Natives and American Indians in Alaska	172	Type: Play	At event	Other Outcomes:	Not reported
					Event Components:		Increased intention to get screened: 37/124 (30% )	
					• Group education			
					• Theatre script for all participants (readers and listeners)			
					Community Guide strategies: GE,SM			
Elmunzer et al. [29]	Colorectal	Post-event only	Uninsured, mostly minority patients older than 50 years who had not undergone CRC screening in the past 10 years in Miami, FL	52	Type: Health fair	At event	Screening:	$6,531
					Event Components:		Flexible Sigmoidoscopy: 52 (100% of attendees; 61.8% of scheduled)	
					Sigmoidoscopy Screening			
					Community Guide strategies: RSB, ROPC			
**Multi-component articles**								
Goldsmith and Sisneros [32]	Breast and cervical	Pre-event and post-event	Migrant and seasonal low income, mostly Hispanic female farmworkers, aged 18+, in California’s Central Valley	1732	Type: Party	45 Days	Screening:	Not reported
					Event Components:		Pap Tests: 183 (10.6%)	
							Mammograms: 29 (1.7%)	
							Clinical Breast Exams: 105 (6.1%)	
					• Group education			
					• Outreach activities (transportation, criteria for free screening and			
					• Scheduling screening appointments)			
					• Screening referrals (vouchers provided)			
					Community Guide strategies: GE, 1 on1,			
					RSB			
Wu et al. [30]	Colorectal	Pre-event and post-event	Mostly uninsured Asian Americans, 50-years or older, at high-risk of colorectal cancer in Michigan	400	Type: Health fair	6-12 mo	Screening:	Not reported
					Event Components:		FOBT: 70 (45%)	
					• Group seminars were on early detection of colorectal cancer		Colonoscopy: 45 (29%)	
							Flexible Sigmoidoscopy: 2 (1%)	
					• Provision of low cost FOBT kits		FOBT and Flexible Sigmoidoscopy: 4 (2%)	
							Other outcomes:	
					• Educational brochures were distributed in all relevant Asian languages		Intention to get screened: 175 (57.6%)	
					Community Guide strategies: GE, RSB			

Note. ^1^Community Guide recommended strategies abbreviations: BC = Breast Cancer Screening, CC = Cervical Cancer Screening, CRC = Colorectal Cancer Screening, ROPC = Reducing Out-of-Pocket Costs, GE = Group Education, 1 on 1 = One-on-One Education, MM = Mass Media, SM = Small Media, RSB = Reducing Structural Barriers. ^2^ Screening numbers for mammograms and CBE were given in aggregate so no percentages could be calculated.

Our literature review identified five types of special events relevant to cancer screening: 1) health fairs [26-30], 2) parties [31,32], 3) a cultural event [33], 4) a special day [34], and 5) a play [35]. Eight studies had the special event as standalone programs while two had the special event along with other educational activities [30,32]. The primary focus of the events varied. Two focused on breast cancer screening only [31,34], three focused on colorectal cancer screening only [29,30,35], three focused both on breast and cervical cancer screening [26,27,32], one focused on breast and colorectal cancer screening [33], and one focused on all three types of screening [28].

The ten special events were designed for diverse populations. The number of participants ranged from 50 to 1,732, with half of the events reporting fewer than 100 participants. Seven events focused on minority populations [29-35], including underserved or underinsured populations. The events were hosted by clinical, academic, or professional organizations, public health agencies, a foundation, and community-based non-profit agencies. Six of the special events reported partnering with community-based non-profits or government agencies to implement the event [27,29,30,32-34]. Two events involved health professionals in-training [27,28], and two employed volunteer health professionals [29,33].

A range of activities were implemented, which were categorized according to the U.S. Community Guide to Prevention Services recommended strategies to increase cancer screening: small media, one-on-one education, group education, reducing structural barriers, and reduced client out-of-pocket cost interventions. Events used two to four strategies; the most common activities involved reducing structural barriers to screening, one-on-one or group education, and provision of cancer educational materials (i.e., small media). Nine events reduced structural barriers to screening, mostly by providing onsite screening [24]; six events employed group education [26,30-33,35]; five events used one-on-one education [26-28,32,33]; five events reduced out-of-pocket costs by offering free or reduced cost breast, cervical, or colorectal cancer screening [27,33,34,29]; and four events offered small media [26,30,34,35]. In addition, three special events provided other health care services including skin or prostate cancer screenings [28,34], health screenings (e.g., hearing, vision) [27], or information on other health issues such as heart health and diabetes [28]. Of those single component special events that offered screening through the program, two special events offered referrals for screening and did not offer onsite screening [30,32].

The special events had different time points and methods for data collection. Seven events collected post-special event data only [26-29,31,34,35], and three collected data pre- and post-special event data [30,32,33]. Seven events collected follow-up data, ranging from 2 days post-event to 12 months post-event [27-34]. Screening outcomes were measured through program or medical records [27-29,31,34], telephone interviews [30-33], and a voucher system at a clinic [32]. Two special events measured other outcomes by surveys regarding the importance of screening, knowledge about cancer screening, and intentions to get screened [35], or scheduling of screening appointments [26]. Follow-up of participants at events that did not offer onsite cancer screening ranged from 48 hours to 12 months [30-33].

Six of the eight single component special events reported numbers of eligible participants attending event who were screened for cancer. The denominator for screening reflects that number of attendees that the events reported in their event/study methods in the articles. Mammography screening rates ranged from 4.8% to 88% [27,33,34], Pap testing was 3.9% [28], and clinical breast exams ranged from 9.1% to 100% [27,28,33]. For colorectal screening, FOBT ranged from 29.4% to 76% [28,33], and sigmoidoscopy was 100% at one event [29]. The one event that focused on breast and colorectal cancer screening reported statistically significant changes in screening from pre-test to post-test (colorectal cancer from 39% to 75% for men and 36% to 76% for women, and mammography from 66% to 84%) [33]. The two multi-component special events also reported cancer screening. Mammography screening was 1.7% [32], Pap testing was 10.6% [32], and screening for colorectal cancer ranged from 1% for flexible sigmoidoscopy to 45% for FOBT [30] at follow-up to the special events. For special events offering onsite screening (n = 4), there were 8–133 clinical breast exams [27,28], 34–44 mammograms [27,34], two Pap tests [28], and 52 sigmoidoscopies [29].

Four of the articles detailed barriers encountered in the implementation of the special event, including recruitment or scheduling the events [31], time management of event coordinators [27], expenses [30], and lack of a screening facility [33]. Three articles provided cost information [27,29,34]. Costs for the special events ranged from $2,500 to $7,000 (mean = $5,343). No cost-effectiveness studies reviewing the cost per person screened were found.

## Discussion

While there are a plethora of research articles on intervention strategies to improve cancer screening rates, this is the first systematic review regarding community special events focused on cancer screening that are designed to increase uptake of breast, cervical, and/or colorectal cancer screening. This study provides a review of published data on the types of events being held, activities and strategies implemented, populations reached and host organizations. In addition, a major contribution of this review is the presentation of a new conceptual framework for linking cancer screening special events to short and long-term outcomes that can be used as a model for examining processes and outcomes associated with such events.

From casual observation, it is widely known that special events are commonplace in our communities. However, our review identified only a handful of published articles that described special events, their components and results. It is likely that articles included in this review represent those events that were better documented and evaluated than most other special events. Still, our review revealed that the methodologies used are mostly descriptive with less rigorous research designs than typically used in the evaluation of other cancer prevention or control interventions or strategies. This is likely due to the service-orientation rather than a research-orientation of these events. It is unclear to the effectiveness of special events in this review due to the variability of the events (i.e., type and combination of cancer screenings) and low rigor of the study designs of the events (e.g., non-experimental designs). In addition, the limited number of articles found and the diversity of special events in terms of type of events and cancer focus make it difficult to determine sufficient evidence for their success.

Despite the small number of articles available for review, our findings suggest that special events are utilizing the evidence-based strategies suggested by the Community Guide. Two common special events combinations found were small media, one-on-one or group education, and reducing structural barriers to screening or education (group or one-on-one) and reducing structural barriers. This is an important finding because it suggests that if deployed widely and with large reach to communities, such events have a real potential to influence uptake of screening. These events can serve as means for outreach for cancer education, particularly for underserved communities that may be harder to reach. Many of the events found focused on minority populations. The number of cancer screenings for the events that were not combined with other activities varied. However, cancer screening can be facilitated, as seen for those special events that offered onsite screening. For example, receipt of clinical breast exams ranged from 6 to 100%. Some differences can be attributed to sample size at the event and capacity or equipment to deliver screening. Reviews of use of one-on-one education intervention found a median post intervention increase was 9.3 percentage points in completed mammography across 31 studies and 8.1 percentage points for pap testing across 8 studies [25]. A recent review of one-on-one education to promote FOBT testing reported a 20.7 median percentage change in screening among 8 untailored interventions [10]. In this review, only one event reported a change in screening of 18% for mammography and 38% for testing by FOBT from baseline to post-event.

Given our findings, this review serves as a call to action for future research efforts to learn more about the nature and effectiveness of these special events since they have the potential for broad population reach (some reaching over 1500 community members). Often community planners of special events may not collect data or publish findings and may require technical assistance for these efforts to advance the field of cancer education. Additionally, special events can draw audiences that might not otherwise have ready access to, or familiarity with, cancer prevention and control services as indicated by the populations being served in the cited review articles. Moreover, these special events are an excellent way to foster stronger relationships across academic, community, and health care sectors and to be mutually beneficial for all stakeholders [36,37].

The next generation of research on cancer screening special events may benefit from attention to elements illustrated in the conceptual framework, beginning with a better appreciation of the conceptual underpinnings linking the event to increased awareness, partnership building, training opportunities, screenings, and improved health outcomes. The field also may benefit from more standardized data elements to be collected at consistent time points, consensus regarding desired short and long-term outcomes, strategies for evaluating cost, and methods for understanding reach (denominator issue). In particular, making data collection instruments easily accessible, such as that being done with the Grid Enabled Measures initiative [38], may enable cross-comparison of events or pooling of data. Further exploration of goals and outcomes of specific types of special events such as health fairs or screening days is warranted.

While this review provides important contributions to the body of knowledge regarding the use and impact of special events, several limitations can be noted. These include publication bias (e.g., few manuscripts of this type published) and restriction to English language, U.S. population, and special events only for specific cancer types. In addition, a formal assessment of study quality was not conducted. This was mainly due to fact that all of the ten articles had evaluations that involved non-experimental designs. The results should be viewed as exploratory since it is difficult to draw conclusions due to limited data published. Also, the variability in types of events that are classified as ‘special events,’ variety of services and activities offered, different settings, target audiences and data collection elements and time points make cross-study comparisons difficult. Thus, as in other health promotion/chronic disease management efforts, future research efforts should identify and clearly delineate essential or “core” components, of screening events that are associated with greater screening rates [39,40]. Similarly, it may be important to move beyond thinking of screening as an isolated discrete event, and to view screening as a dynamic process that involves initial education and continual follow-up activities. Also, this review suggests that to gain a fuller understanding of the nature, scope and potential reach of special cancer screening events, professionals may need to look beyond articles published in peer-reviewed journals and additionally focus efforts on capturing information from the “grey literature”, namely conference presentations at the local and national level. Finally, a meta-analysis of the fındings was considered early in the process, but this analytical approach was deemed inappropriate due to the general lack of outcome data elements pre- to post-intervention or inclusion of a comparison group (e.g., non-experimental). Instead, the findings of the review describe available information regarding the demographics of the population served, type of special event, type of cancer screening, components of the event, cost information and other relevant data collected.

## Conclusions

This review found that health fairs and parties were the most common special events. Many events offered one on one or group education, health education materials, and reduced or free cancer screening. This review found that there is insufficient evidence that special events promote cancer screening. This is mostly due to the limited data presented or lack of rigorous designs of these events (e.g., posttest only). However, some events had high screening rates through onsite screening such as 88% for mammograms or 100% for colonoscopies; however, their reach was relatively low (less than 60). While cancer prevention and control interventions have traditionally been evaluated by ‘gold standard’ randomized clinical trial designs, a better understanding of the nature, processes, and outcomes of special events calls for an appreciation of other more pragmatic evaluation designs since this is a new field and methods are evolving [41]. Further investigations of special events to increase cancer screening would provide further knowledge about their effects. In addition, research is needed to strengthen the evidence base of specific types of special events such as health fairs or cultural festivals.

Despite the noted research limitations, this first comprehensive, systematic review of cancer screening events has advanced knowledge of cancer prevention and control by the development of a conceptual model, abstraction of multiple data elements (participants, intervention strategies, outcomes), and recommendations for standardizing/improving future reporting of findings. There is a critical need for evaluation of these community events to define their effectiveness due to the limited numbers of events published. The development of common outcomes measures and encouraging of professionals in the communities who implement these events to evaluate them would contribute to practice–based evidence. Partnering with universities or other organizations with evaluation expertise would also develop the capacity of local health professionals to examine the benefits of these special events for promoting cancer screening. Through further evaluations, the effectiveness of special events as a promising intervention for promoting cancer screening will be better understood.

## Competing interests

The authors declared that they have no competing interests.

## Authors’ contributions

CE, K Rodgers, MK, DH, K Roland and RH participated in the design of the study. CE, KR, EP, MK, DH and SL carried the systematic review and reviewed the studies. CE, K Rodgers, EP, SL, JA, MF, MO, RB, BR, and TB helped to draft the manuscript. All authors read and approved the final manuscript.

## Pre-publication history

The pre-publication history for this paper can be accessed here:

http://www.biomedcentral.com/1471-2458/14/274/prepub

## Supplementary Material

Additional file 1 Data Abstraction Form. Click here for file
